# Minor effect of inaccurate fixation on VEP-based acuity estimates

**DOI:** 10.1007/s10633-020-09796-7

**Published:** 2020-10-10

**Authors:** Amal A. Elgohary, Sven P. Heinrich

**Affiliations:** 1 Department of Vision Science, Research Institute of Ophthalmology, Giza, Cairo, Egypt; 2 Eye Center, Medical Center, University of Freiburg, Killianstr. 5, 79106 Freiburg, Germany; 3 Faculty of Medicine, University of Freiburg, Freiburg, Germany

**Keywords:** Objective acuity testing, Visual evoked potentials, Eccentric fixation, Visual acuity, malingering, functional impairment

## Abstract

**Purpose:**

VEP-based estimation of visual acuity may be used in cases of suspected malingering to objectify subjective complaints. In such an application, a lack of cooperation needs to be expected. The same may apply to young children with suspected functional impairments. In the present study, we assessed how inaccurate fixation affects the acuity estimates obtained with a VEP technique.

**Methods:**

VEP-based acuity estimates were obtained by stimulating with a series of different check sizes using a ‘stepwise sweep’ protocol. Sixteen participants were tested with normal and degraded vision under five different fixation conditions (central fixation and eccentric fixation at top, bottom, right, and left edge of the stimulus area).

**Results:**

The majority of individual acuity estimates with eccentric fixation differed by less than 0.1 logMAR from central fixation, and almost all estimates differed by less than 0.3 logMAR. Median estimates with eccentric fixation differed only slightly (up to 0.08 logMAR) and, except for top fixation with normal vision, non-significantly. However, data quality was lower with eccentric fixation, which increased the probability that no acuity estimate could be derived from the recording.

**Conclusion:**

VEP-based acuity estimates are relatively insensitive to eccentric fixation. Unnoticed deviations from central fixation in routine applications will probably be smaller than in the present study and will have even less impact on the outcome.

## Introduction

VEP-based estimation of visual acuity is increasingly popular as a technique in cases of suspected malingering or when a patient is unable to cooperate during standard psychophysical acuity testing [1]. Within certain limits, the reliability of the approach has been demonstrated in a number of studies (e.g., [2–4]), except with disorders that are associated with distortions of the visual percept, such as amblyopia [5, 6] and probably also when the reason for the impairment has a locus beyond the primary visual cortex.

During VEP measurements, patients are normally asked to fixate the center of the screen, where a fixation mark is usually provided. Given that suspected malingering is one of the prime scenarios for the use of VEP-based acuity estimation, it is an important question whether incorrect fixation leads to a sizable effect on the outcome of the test. In addition to typical cases of malingering as encountered in the normal clinical routine, misrepresentation of visual impairments by athletes in the classification of participants in vision impaired sports [7] is another potential field of application of VEP-based techniques. Furthermore, incorrect fixation may also occur in young children and patients with mental disabilities or certain oculomotor disorders.

While the problem may in principle be ameliorated by increasing the total stimulus size, this is often not feasible due to technical limitations. For instance, trying to achieve this by moving the screen closer to the patient will increase the angular size of the pixels and thus limit the range of visual resolutions that can be tested.

The present study aimed at addressing this question by obtaining VEP-based acuity estimates from healthy cooperative participants with normal and artificially reduced acuity who are instructed to fixate eccentrically.

## Methods

### Participants

Sixteen normal participants (age range 23–38 years) with normal or corrected-to-normal visual acuity (logMAR ≤ 0.0 as measured with the Freiburg Acuity and Contrast Test (FrACT) [8]) participated in the study. All reported free of ophthalmological or neurological disorders and provided written informed consent. The study had been approved by the local institutional review board at the University of Freiburg and followed the tenets of the Declaration of Helsinki. The eye with better visual acuity, or the right eye when both eyes had equal acuity, was selected as study eye.

### Stimuli and procedure

VEP-based estimation of visual acuity is not covered by the standard ISCEV VEP standard [9], and an ISCEV extended protocol [10] was only published very recently. We used the procedure described in detail by Bach et al. [11], which is within the specifications of the ISCEV extended protocol. In short, checkerboard onset stimuli were displayed monocularly at a frequency of 7.5 Hz on a CRT monitor with a Michelson contrast of 40% and a frame rate of 75 Hz. The size of the stimulus area was 19° × 15°. Six logarithmically approximately equidistant check sizes of 0.046° to 0.37° were used. The observation distance was 114 cm. Participants were supplied with a near addition to adjust the refraction to the monitor distance. They were tested with normal vision and with vision artificially blurred using Bangerter occluders (grade 0.4). Five different fixation conditions, namely central, right, left, top and bottom, were tested. The order of the 10 different conditions (5 fixation locations × 2 acuity levels) was randomized individually for each participant. The VEPs were recorded with a Laplacian electrode montage (Oz versus (O1 + O2)/2).

Eccentric fixation was achieved by asking the participant to fixate a target that was attached to the edge of the stimulus area on the respective side, so that the horizontal or vertical meridian of the visual field aligned with the edge of the stimulus area. For instance, for left eccentric fixation, the fixation target was in the middle of the left edge of the stimulus area, such that the vertical meridian of the visual field coincides with the edge of the stimulus area and stimulation was limited to the right hemifield.

### VEP analysis and acuity estimation

Following Bach et al. [11], all artifact-free 1-s intervals of the steady-state response to a given check size were averaged, and Fourier analysis was applied to extract the response at the stimulation frequency (7.5 Hz). A noise correction was applied to the amplitudes [12], and response significance was determined [13]. Because in the degraded vision condition most check sizes were too small to be resolved, the chance of spurious significances increased (multiple testing problem). In a few cases, we therefore had to manually correct for this by treating the respective data points as not having a statistically significant response.

The resulting tuning curve (amplitude vs. spatial frequency) was extrapolated to zero amplitude using Bach et al.’s [11] heuristic algorithm. The corresponding spatial frequency SF_0_ was then converted into an acuity estimate using an empirically determined conversion factor that had been determined by that same study using the relationship logMAR = log(17.6/SF_0_) [11].

### Statistical assessment

The present study assessed a diagnostic procedure that is usually applied to individual persons. Consequently, the question of statistical significance on the group level was not the primary concern. For completeness, however, we performed pairwise comparisons between the acuity estimates obtained with eccentric fixation and the corresponding acuity estimate obtained with central fixation to identify those eccentric fixation locations where the pattern of effects is consistent enough to result in a significant effect on the group level. This was done by applying permutation tests that do not rely on the assumption of a normal distribution [14]. Confidence intervals for differences in acuity estimates were obtained through bootstrapping [14].

## Results

**Fig. 1:**
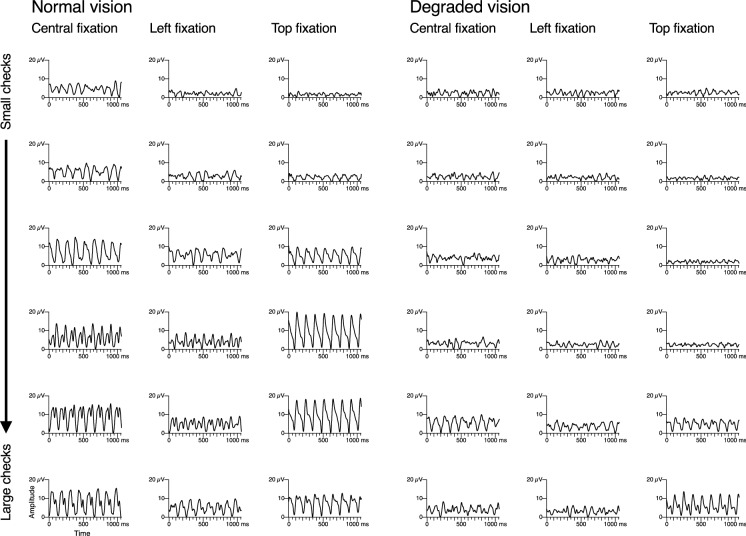
Sample traces of one participant, obtained with central, left and top fixation. Check size increases from top to bottom. For large checks with normal vision, responses are clearly recognizable. With smaller checks or with degraded vision, responses are reduced or absent. What looks like a response to the smallest checks in some traces is in fact not a 7.5-Hz stimulus response, but an unrelated oscillation at a higher frequency, most likely alpha activity [15]. As an interesting side detail (unrelated to the topic of the present study), a superposition of the presumed alpha activity and the stimulus response probably underlies the beating waves in some traces. Tuning curves of the same participant are shown in the top set of graphs in Fig. 2

**Fig. 2:**
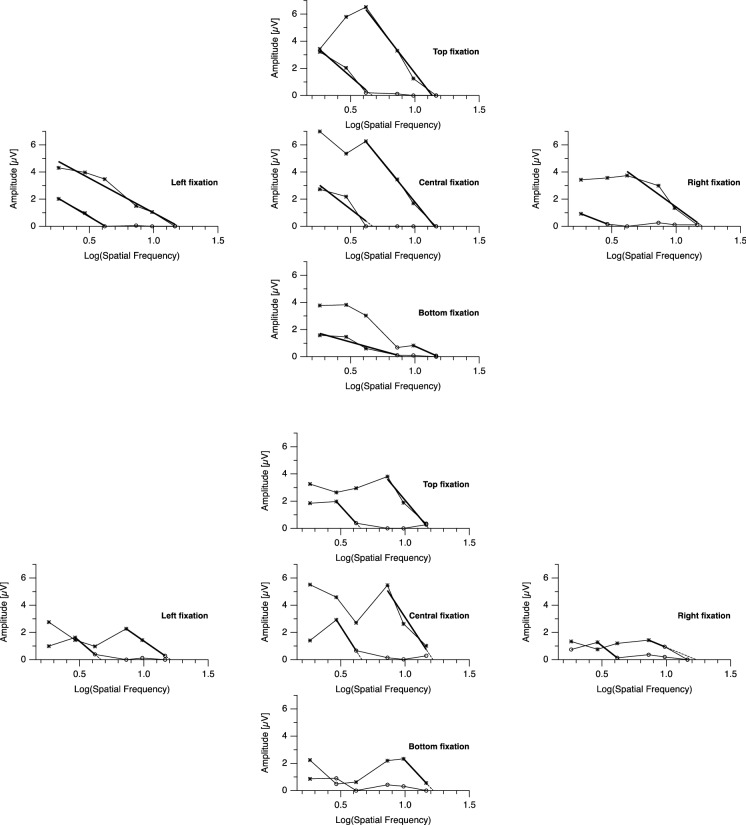
Comparison of tuning curves. Each group of five graphs shows data of all five fixation conditions of one sample participant. Within each graph, the top curve was obtained with normal vision, and the lower curve with degraded vision. Asterisk markers indicate statistically significant responses. Bold lines represent straight lines fitted to the descending slope of the tuning curve. Dashed lines show the extrapolation to the abscissa, with the intercept yielding the threshold estimate. With normal vision, eccentric fixation typically reduces the amplitudes while leaving the threshold largely unchanged. This is similar in degraded vision, although variability is higher and in some cases the algorithm may fail to yield and acuity estimate (lower set of graphs, bottom graph)

As shown in Fig. 1 for a sample participant, VEP responses with eccentric fixation were generally smaller than with central fixation, albeit with exceptions in particular with top fixation. With normal vision, in two out of 80 recordings (16 participants × 5 fixation locations), no acuity estimate could be obtained. With degraded vision, the respective number was 14. Generally, visual inspection suggests that data quality was lower with eccentric fixation (see example in Fig. 2).

We compared the participants’ standard acuity under normal vision conditions as measured with the FrACT to the respective VEP-based acuity estimates obtained with central fixation. In the majority of participants, both agreed within the limits reported by Bach et al. [11]. For six participants (5 of them with a logMAR value below − 0.3, i.e., very good acuity substantially beyond the range covered by the VEP stimuli), acuity with normal vision was underestimated by the VEP method.

**Fig. 3:**
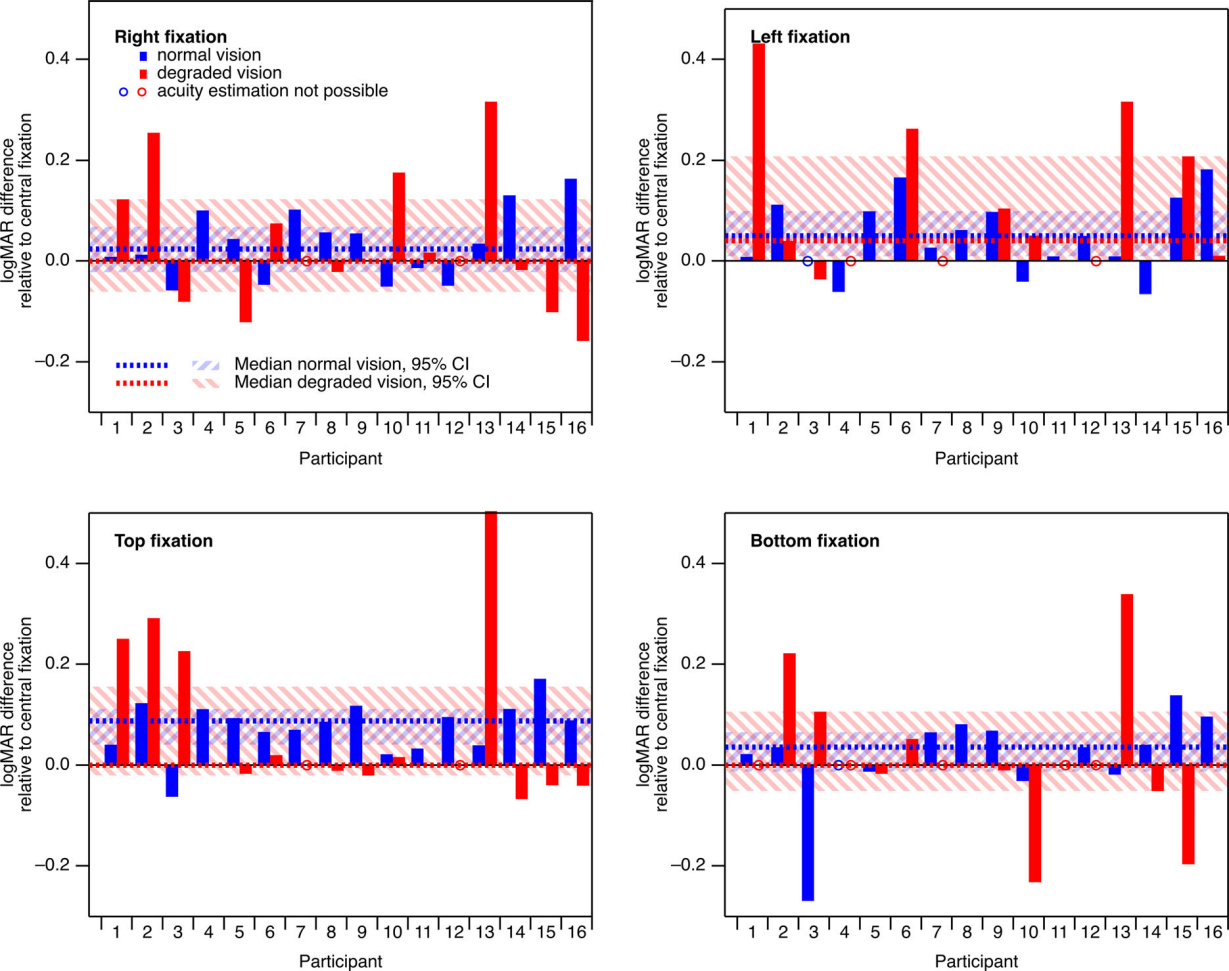
Effect of eccentric fixation on acuity estimate in individual participants. In both normal vision (blue) and degraded vision (red), the median effects (dotted lines) were quite small and, with the exception of top fixation with normal vision, not significant

Figure 3 provides an overview of logMAR differences between VEP-based estimates of eccentric and central fixation for all individual participants. With normal vision, the absolute logMAR difference was below 0.1 in 75% of the successful acuity estimates, with the remainder below 0.3. With degraded vision, the absolute logMAR difference was below 0.1 in 60% of the successful acuity estimates and below 0.3 in another 31%.

On the group level, in normal vision, the median logMAR differences with right, left, top and bottom eccentric fixation were 0.02 (95% CI, −0.02…0.06; *p* = 0.09), 0.05 (0.01…0.09; *p* = 0.02), 0.08 (0.04…0.11; *p* < 0.001) and 0.03 (−0.01…0.06; *p* = 0.53), respectively. In degraded vision, the median logMAR differences with right, left, top and bottom eccentric fixations were 0.00 (95% CI, −0.06…0.12; *p* = 0.41), 0.04 (0.00…0.21; *p* = 0.01), 0.00 (−0.02…0.15; *p* = 0.12) and 0.00 (−0.05…0.11; *p* = 0.70), respectively. Only the effect with top fixation and normal vision was significant with a Bonferroni correction.

## Discussion

In this study, the employed four eccentric fixations were chosen such that stimulation was limited to one half of the visual field in each condition. Stimuli extended from the respective meridian to moderate eccentricities as defined by the size of the stimulus field.

The median increase in logMAR estimates was in the range of 0.02–0.08 for normal vision and 0.00–0.04 for degraded, which (in the absence of knowledge about incorrect fixation) would for most eccentric fixation conditions imply a slightly worse acuity, albeit only statistically significant for top fixation with normal vision. The magnitude of the median effect was less than one line on an acuity chart and thus not very relevant in most cases in which VEP-based acuity estimated would be obtained routinely in individual patients. For comparison, the International Council of Ophthalmology proposes that a mean difference of 0.05 should be taken as the limit for accepting two acuity tests as being equivalent [16]. The situation might be different in group comparisons, as opposed to the assessment of individual patients, if the fixation behavior between groups differs, potentially resulting in a misleading finding of a statistically significant (albeit small) acuity difference.

The median increase in logMAR estimates does not represent the full picture, though. As the examples in Fig. 3 illustrate, there is a considerable interindividual variability. However, in the majority of individual acuity estimates, the absolute logMAR difference was below 0.1, and in almost all cases it was below 0.3. The latter value is the approximate range within which VEP-based estimates match psychophysical acuity in 95% of the cases [11].

Importantly, the effects may have either direction (higher or lower values than with central fixation). In our data set, this is particularly obvious with bottom fixation. A large contribution to these effects is likely to come from general measurement variability rather than from eccentric fixation. The lower data quality with eccentric fixation probably also increases the risk of occasional ‘outliers’ and explains why no acuity estimate could be obtained in a number of recordings with eccentric fixation.

The use of a Laplacian montage probably contributed beneficially to the study outcome as it is known to yield a better signal-to-noise ratio than a recording from Oz only (referenced, for instance, to a frontal electrode) [17]. Especially with right or left fixation, partly depending on the individual cortical structure, selecting only the Oz–O1 or Oz–O2 bipolar derivations from the Laplacian arrangement may yield further improvement [18].

The present findings are not unexpected, of course. The central visual field has a disproportionally large contribution to the VEP response [19]. In addition, especially with undegraded vision, near-threshold stimuli can only be resolved in the central visual field where acuity is highest. Thus, when the edge of the stimulus is fixated, the effective amount of neuronal stimulation near threshold can be expected to be halved or even further reduced. However, the effect on the VEP might be quite different, as evident from the fact that the responses may even increase with top fixation (Fig. 1). This is because the folding of the visual cortex, in particular at the calcarine sulcus, has a strong effect on the amount of activation that is picked up by an electrode and may result in a polarity inversion for some VEP components when stimulation is switched between the upper and lower visual field [18, 20]. A visual inspection of the time course data across participants suggests a large variability of the VEP traces in this respect. In some participants, there was a polarity inversion between top and bottom fixation, which was absent in other participants. In some cases, the curve shapes differed in a way that cannot simply be described in terms of polarity or the effect depended on the check size. These differences may partly explain interindividual variability in the amount of amplitude change with eccentric fixation, particularly in the case of top and bottom fixation. While a general amplitude change should not have a sizable effect on the threshold of the VEP tuning curve, a dependence on check size could affect the outcome.

Given the stimulus dimensions in the present study, fixating the edge of the stimulus area results in a deviation from central fixation of about 7.5° vertically or 9.5° horizontally. This amount is large enough to be spotted by an experienced technician. It seems safe to assume that any deviation that is smaller and goes unnoticed will have less impact on the results than found in the present study. Thus, incorrect fixation will have little effect on acuity estimates. The present findings may also facilitate the interpretation of test outcomes in patients with eccentric fixation due to central visual field loss, for instance when using a preferred retinal locus [21].

In summary, the present data suggest that VEP-based acuity estimates are relatively insensitive to deviations from central fixation.

## Data Availability

Data are available from the corresponding author upon reasonable request.
